# Obituary for Prof. Dr. Johng Sik Rhim 

**DOI:** 10.20517/evcna.2022.15

**Published:** 2022-03-25

**Authors:** EVCNA Editorial Office

**Affiliations:** Suite 1504, Tower A, Xi’an National Digital Publishing Base, No. 996 Tiangu 7th Road, Gaoxin District, Xi'an 710077, Shaanxi, China

With deep sadness, we announce that our Editorial Board member Johng Sik Rhim passed away peacefully on March 5, 2022, in Bethesda, MD, USA. 

Dr. Rhim is a pioneer in human cell transformation whose career has been suffused with six decades of contributions to the field of oncology and cancer research. His interest in medicine developed after a grueling six-year battle with septic arthritis, beginning when he was just three years old, and he earned a Doctor of Medicine from Seoul National University College of Medicine in 1957. Dr. Rhim completed a medical internship at Seoul National University Hospital through the beginning of 1958, and relocated to the United States to accept a two-year research fellowship at Dr. Albert B. Sabin’s laboratory at the Children’s Hospital Research Foundation later that year.

Throughout the early 1960s, Dr. Rhim would pursue research fellowships and associate positions with Baylor University College of Medicine, the Graduate School of Public Health at the University of Pittsburgh, and Louisiana State University School of Medicine. In 1964, he joined the National Institute of Allergy and Infectious Diseases at the National Institutes of Health as a visiting scientist. Dr. Rhim subsequently departed in 1966 to become the project director for Cancer Research at Microbiological Associates of Bethesda, Maryland.

In 1978, Dr. Rhim was named as a senior investigator at the National Cancer Institute, a part of the National Institutes of Health. He would remain active with the National Cancer Institute until 1998, whereupon he accepted his current role as the Associate Director, Center for Prostate Disease Research, Research Professor Emeritus, Department of Surgery, at the Uniformed Services University of the Health Sciences. Dr. Rhim’s research has focused on the development of human cell culture systems for use in cancer studies, which have allowed for new in-depth analysis of the mechanisms of action of therapies and carcinogens. He holds many patents for processes related to cell transformation, and looks forward to furthering advances in patient-derived cell models based on his early models. As a pioneer in human cell modeling and cultures, Dr. Rhim has contributed more than 300 articles and maintained involvement with numerous professional societies, including the American Medical Association, the American Association for the Advancement of Science, the Society of Experimental Biology and Medicine, and the International Association of Leukemia Research^[1]^.

Johng Sik Rhim was Editorial Board member of *Extracellular Vesicles and Circulating Nucleic Acids*. He supported the peer-review for *EVCNA*, and we highly appreciate his support to *EVCNA* at its early stage.

## DECLARATIONS

### Copyright

© The Author(s) 2022.
